# Supplementary material for: Research on emergency material demand based on urgency and satisfaction under public health emergencies
Source: PLoS One. 2023 Mar 23;18(3):e0282796. doi: 10.1371/journal.pone.0282796 (PMC10035926; doi:10.1371/journal.pone.0282796)
Supplement: S1 File — (DOCX) [file pone.0282796.s001.docx]

Table 1. Infected people

| Date | Infected people |
| --- | --- |
| 2022.4.05 | 83 |
| 2022.4.06 | 81 |
| 2022.4.07 | 69 |
| 2022.4.08 | 67 |
| 2022.4.09 | 54 |
| 2022.4.10 | 31 |
| 2022.4.11 | 20 |

The data comes from the Hebei Provincial Health Commission (2022.04.05–04.11, <http://wsjkw.hebei.gov.cn/>).

Table 2.Results of the demand side vote

|  | Demander 1 | Demander 2 | Demander 3 | Demander 4 | Demander 5 |
| --- | --- | --- | --- | --- | --- |
| Index attribute 1 | 0.09 | 0.083 | 0.089 | 0.102 | 0.105 |
| Index attribute 2 | 0.11 | 0.15 | 0.092 | 0.047 | 0.108 |
| Index attribute 3 | 0.065 | 0.058 | 0.083 | 0.075 | 0.092 |
| Index attribute 4 | 0.042 | 0.039 | 0.035 | 0.016 | 0.028 |
| Index attribute 5 | 0.085 | 0.091 | 0.105 | 0.095 | 0.103 |
| Index attribute 6 | 0.13 | 0.15 | 0.073 | 0.18 | 0.105 |
| Index attribute 7 | 0.143 | 0.131 | 0.135 | 0.14 | 0.098 |
| Index attribute 8 | 0.13 | 0.11 | 0.153 | 0.112 | 0.132 |
| Index attribute 9 | 0.15 | 0.145 | 0.124 | 0.115 | 0.104 |
| Index attribute 10 | 0.055 | 0.043 | 0.111 | 0.118 | 0.134 |

The voting weight of the 5 demanders on the index attribute comes from the **Questionnaire 1**.

Table 3. Results of the supply side vote

|  | Supplier 1 | Supplier 2 | Supplier 3 | Supplier 4 | Supplier 5 |
| --- | --- | --- | --- | --- | --- |
| Index attribute 1 | 0.311 | 0.285 | 0.293 | 0.269 | 0.258 |
| Index attribute 2 | 0.258 | 0.263 | 0.294 | 0.238 | 0.231 |
| Index attribute 9 | 0.209 | 0.225 | 0.233 | 0.242 | 0.245 |
| Index attribute 10 | 0.222 | 0.227 | 0.225 | 0.251 | 0.266 |

The voting weight of the 5 suppliers on the index attribute comes from the **Questionnaire 1**.

Table 4. The supplier's evaluation of material requirements urgency

| $L_{n}$ | $C_{1}$ | $C_{2}$ | $C_{3}$ | $C_{4}$ | $C_{5}$ | $C_{6}$ | $C_{7}$ | $C_{8}$ | $C_{9}$ | $C_{10}$ |
| --- | --- | --- | --- | --- | --- | --- | --- | --- | --- | --- |
| $L_{1}$ | 3 | 4 | 1 | 2 | 2 | 2 | 3 | 2 | 2 | 4 |
| $L_{2}$ | 5 | 1 | 3 | 2 | 1 | 1 | 3 | 3 | 2 | 4 |
| $L_{3}$ | 2 | 2 | 2 | 4 | 3 | 3 | 4 | 2 | 3 | 3 |
| $L_{4}$ | 4 | 3 | 2 | 5 | 5 | 2 | 2 | 4 | 3 | 3 |
| $L_{5}$ | 1 | 3 | 4 | 5 | 4 | 1 | 3 | 1 | 4 | 2 |

The evaluation value of the index attribute by suppliers comes from the **Questionnaire 1**.

$L_{n}$: suppliers; $C_{l}$: index attributes;

Table 5. The demander's expectation of the urgency of the material requirements

| $T_{n}$ | $C_{1}$ | $C_{2}$ | $C_{3}$ | $C_{4}$ | $C_{5}$ | $C_{6}$ | $C_{7}$ | $C_{8}$ | $C_{9}$ | $C_{10}$ |
| --- | --- | --- | --- | --- | --- | --- | --- | --- | --- | --- |
| $T_{1}$ | 3 | 2 | 2 | 3 | 2 | 4 | 1 | 4 | 3 | 2 |
| $T_{2}$ | 2 | 1 | 3 | 3 | 2 | 4 | 3 | 5 | 2 | 2 |
| $T_{3}$ | 2 | 2 | 1 | 2 | 1 | 3 | 2 | 3 | 4 | 3 |
| $T_{4}$ | 2 | 3 | 2 | 2 | 1 | 4 | 2 | 1 | 4 | 4 |
| $T_{5}$ | 1 | 3 | 4 | 4 | 3 | 4 | 3 | 2 | 3 | 4 |

The evaluation value of the index attribute by demanders comes from the **Questionnaire 1**.

$T_{n}$: demanders; $C_{l}$: index attributes;

We use GA code to solve the bi-objective model.

GA code:

clear all;

close all;

clc;

global vb vs rmat

vb=[0.1015 0.1084 0.0627 0.0688 0.1135

0.1063 0.0713 0.0842 0.1145 0.1165

0.0723 0.0827 0.0676 0.0980 0.1075

0.0724 0.0939 0.0803 0.1055 0.0941

0.0987 0.1147 0.0487 0.0869 0.0540];

vs=[ 0.1488 0.1628 0.0441 0.0625 0.0505

0.1285 0.1100 0.0575 0.1027 0.0542

0.1284 0.1441 0.0496 0.0961 0.0585

0.0813 0.1337 0.0962 0.1061 0.0656

0.0961 0.1503 0.0607 0.0723 0.0593];

rmat=[3 4 1 2 2 2 3 2 2 4

5 1 3 2 1 1 3 3 2 4

2 2 2 4 3 3 4 2 3 3

4 3 2 5 5 2 2 4 3 3

1 3 4 5 4 1 3 1 4 2];

nvar = 25;

% rng default % For reproducibility

zero25 = zeros(25,1);

ind = reshape(1:25,5,5);

eq1 = zero25;

eq1(ind(1,:)) =1;

eq2 = zero25;

eq2(ind(2,:)) =1;

eq3 = zero25;

eq3(ind(3,:)) =1;

eq4 = zero25;

eq4(ind(4,:)) =1;

eq5 = zero25;

eq5(ind(5,:)) =1;

eq6 = zero25;

eq6(1:5) =1;

eq7 = zero25;

eq7(6:10) =1;

eq8 = zero25;

eq8(11:15) =1;

eq9 = zero25;

eq9(15:20) =1;

eq10 = zero25;

eq10(21:25) =1;

myfun = @myobj;

A= [eq6,eq7,eq8,eq9,eq10]';

b = [1;1;1;1;1];

Aeq = [eq1,eq2,eq3,eq4,eq5]';

beq = [1;1;1;1;1];

% 定义域

intcon = 1;

lb = [zeros(25,1)];

ub = [ones(25,1)];

nonlcon = @nonlcon;

options = optimoptions("ga",'PopulationSize',10,'CrossoverFraction',0.8,'TolFun',1e-100,...

'MutationFcn', {@mutationadaptfeasible}, 'MigrationFraction',0.2,'Generations',100,'StallGenLimit',100 ,...

'PlotFcns',{@gaplotbestf,@gaplotexpectation });

[x,fval,exitflag,output,population,scores] = ga(myfun,nvar,A,b,Aeq,beq,lb,ub,[],intcon,options);

% 目标函数值还原到原始问题

f = -fval;

% 优化方案

xresult=reshape(x,5,5);

Z1 = reshape(vb,25,1).*x';

Z2 = reshape(vs,25,1).*x';

obj1 =sum(Z1)

obj2 =sum(Z2)

%%%%%%%%%%%%%%%%%%%%%%%%%%%%%%%%

function my_obj = myobj(x)

load vb;

load vs;

load rmat

r =rmat(1:5,1:5);

Z1 = reshape(vb,25,1).*x';

Z2 = reshape(vs,25,1).*x';

Z3 = reshape(r,25,1).*x';

my_obj = -0.5.*(sum(Z1)+sum(Z2));

% my_obj = -0.4.*(sum(Z1)+sum(Z2))-0.2.*sum(Z3);

End

%%%%%%%%%%%%%%%%%%%%%%%%%%%%%

function mycon = nonlcon(x)

zero25 = zeros (25,1);

eq1 = zero25;

eq1(1:5) =1;

eq2 = zero25;

eq2(6:10) =1;

eq3 = zero25;

eq3(11:15) =1;

eq4 = zero25;

eq4(16:20) =1;

eq5 = zero25;

eq5(21:25) =1;

[sum(x(eq1))-1;sum(x(eq2))-1;sum(x(eq3))-1;sum(x(eq4))-1;sum(x(eq5))-1];

End

**Questionnaire 1**

**Managing and scheduling emergency logistics is very important in the Public Health Emergency environment. In order to better understand the supply-demand matching problem by considering the factors that affect the urgency and satisfaction of the demand for emergency supplies, we have done the following survey.**

1. **Your gender is:**

- **Man□**
- **Woman□**

1. **What is the distance between your community location and the supply point?**

- **<500** **meters□**
- **500-1000** **meters□**
- **1000-1500** **meters□**
- **1500-2000** **meters□**
- **>2000** **meters□**

1. **What is the supply capacity?**

- **Supply capacity is the weakest□**
- **Supply capacity is weak□**
- **Supply capacity is average□**
- **Supply capacity is strong□**

**Supply capacity is the strongest□**

1. **How many people are in difficulty (old, weak, sick, disabled, pregnant) in your family?**

- $\geq$**5** **people□**
- **4-5** **people□**
- **3-4** **people□**
- **2-3** **people□**
- $\leq$**1** **people□**

1. **The total number of your family members is:**

- $\geq$**5** **people□**
- **4-5** **people□**
- **3-4** **people□**
- **2-3** **people□**
- **1-2** **people□**

1. **The number of people affected by a public health emergency in your home is:**

- $\geq$**5** **people□**
- **4-5** **people□**
- **3-4** **people□**
- **2-3** **people□**
- **1-2** **people□**

1. **The number of days your emergency reserve is available is:**

- $\geq$**15** **days□**
- **10-15** **days□**
- **5-10** **days□**
- **1-5** **days□**
- **<1 day□**

1. **At present, your non-replaceable items are out of stock for:**

- **<1 day□**
- **1-3** **days□**
- **3-5** **days□**
- **5-7** **days□**
- $\geq$**7** **days□**

1. **The distance from your location to the transportation hub is:**

- **<500米□**
- **500-1000** **meters□**
- **1000-1500** **meters□**
- **1500-2000** **meters□**
- $\geq$**2000** **meters□**

1. **Your current delivery fee is:**

- **The cost of distribution is highest□**
- **The cost of distribution is higher□**
- **The cost of distribution is moderate□**
- **The cost of distribution is lower□**
- **The cost of distribution is lowest□**

1. **What is your current delivery time?**

- **The distribution time is shortest□**
- **The distribution time is shorter□**
- **The distribution time is moderate□**
- **The distribution time is longer□**
- **The distribution time is longest□**

1. **Which of the current indicators do you think has the most impact on the urgency of material needs?**

- **Location□**
- **Supply capacity□**
- **Number of needy family members□**
- **Total number of family members□**
- **Number of families affected□**
- **Status of emergency supplies□**
- **Non-replaceable items out of stock for a long time□**
- **Transportation convenience□**
- **Distribution cost□**
- **Delivery time□**
